# Emergency for dermatologic wards?

**DOI:** 10.1111/jdv.20619

**Published:** 2025-04-25

**Authors:** Falk Ochsendorf

**Affiliations:** ^1^ Goethe University Frankfurt, University Hospital Clinic for Dermatology, Venereology and Allergy Frankfurt am Main Germany

The study ‘Dermatological emergencies and determinants of hospitalization in Switzerland: a retrospective study’^1^ in this issue sheds light on a very relevant practical point: how do patients with acute dermatological problems leading to hospitalization receive optimal care? In this study, 79% of patients who presented to an emergency department with a major dermatological problem after initial assessment were admitted to hospital—but only 2.2% were admitted to a dermatology ward. This and other findings of the study raise numerous questions.

1. Can this result be transferred to other clinics, or is it the result of a local organizational peculiarity in the authors' institution? In the latter case, it could only be discussed locally whether this situation is the result of political/administrative decisions or whether this approach is really the optimal care pathway in terms of the best possible patient care. However, if this result reflects a general trend, then the question arises as to how the specialist societies should react if obviously clear dermatological problems are not primarily treated on the dermatology wards best suited for this. This would then require a broad discussion and correspondingly coordinated measures. For Germany, a consistently high demand for inpatient care of dermatological patients was described,^2^ but the data (figure 1) in the aforementioned study^1^ could indicate a marginalization of inpatient dermatology.
2. It is noticeable that in this study^1^ so many patients who presented with dermatological emergencies in the emergency department required inpatient care. This rate was significantly lower in other settings. As an example, a retrospective study from Turkey found only 11% inpatient admissions following dermatology consultations in paediatric and general emergency departments.^3^ In this respect, local care pathways appear to have a decisive influence.
3. Comparative studies on the treatment outcomes of patients with the same dermatological conditions treated either on a dermatology ward or on another ward are not available (query at ‘Pubmed’ and ‘Perplexity’ 20 February 2025). In order to prove that care on a dermatology ward is more beneficial, studies with endpoints such as length of stay, resource consumption or complication rates would be conceivable and desirable.
4. The relevance of adequate dermatological nursing care for such patients has obviously not been adequately considered to date. In patients with sepsis, it was shown that care by specialized nursing staff shortened the length of stay and led to significant cost savings.^4^ Corresponding studies on dermatologically trained nurses and their impact on treatment outcomes are currently lacking. An older review of the benefits of providing patient care with dermatologically trained nurses pointed to generally positive effects. However, the studies had methodological weaknesses.^5^ This aspect therefore remains an under‐researched area.
5. The variables found in the descriptive statistics,^1^ which are intended to better identify the risk of inpatient treatment in a dermatological emergency, would be helpful for an initial assessment at the acute presentation of such patients. However, their actual suitability would first have to be prospectively tested and validated in an independent collective. It is conceivable that optimized treatment pathways could be derived from this.

In this respect, the above‐mentioned work^1^ encourages numerous further investigations to clarify the open questions mentioned. Above all, however, it is a wake‐up call for all hospitals with dermatology departments to review how patients with acute dermatological conditions are directed from the emergency department to the wards providing care. This is the only way to ensure that these patients receive optimal medical and nursing care on a dermatology ward.

## CONFLICT OF INTEREST STATEMENT

None.

## Data Availability

Data sharing not applicable to this article as no datasets were generated or analysed during the current study.
